# Tandem Osmotic Engine Based on Hydrogel Particles with Antipolyelectrolyte and Polyelectrolyte Effect Fuelled by Both Salinity Gradient Modes

**DOI:** 10.3390/gels7040232

**Published:** 2021-11-25

**Authors:** Anjali Cheeramthodi Padmanabhan, Dong Suk Han, Sifani Zavahir, Jan Tkac, Peter Kasak

**Affiliations:** 1 Center for Advanced Materials, Qatar University, Doha P.O. Box 2713, Qatar; anjali.acp4@gmail.com (A.C.P.); dhan@qu.edu.qa (D.S.H.); fathima.z@qu.edu.qa (S.Z.); 2 Department of Chemical Engineering, College of Engineering, Qatar University, Doha P.O. Box 2713, Qatar; 3 Institute of Chemistry, Slovak Academy of Sciences, Dubravska Cesta 9, 84538 Bratislava, Slovakia; jan.tkac@savba.sk

**Keywords:** energy recovery, hydrogel, salinity gradient energy, antipolyelectrolyte effect, polyelectrolyte effect

## Abstract

In this study, we propose a new approach to attain energy by salinity gradient engines with pistons based on hydrogels possessing polyelectrolyte and antipolyelectrolyte effects in a tandem arrangement, providing energy in each salinity gradient mode in a repeatable manner. The swelling of hydrogel with a polyelectrolyte effect and shrinking of hydrogel particles possessing an antipolyelectrolyte effect in desalinated water, and subsequent shrinking of hydrogel with polyelectrolyte and swelling of hydrogel antipolyelectrolyte effect in saline water, generate power in both increasing and decreasing salinity modes. To investigate the energy recovery, we scrutinized osmotic engine assemblies by a setup arrangement of pistons with hydrogel particles, with polyelectrolyte and antipolyelectrolyte effects, in tandem. The energy recovery from the tandem engine setup (calculated based on dry form for each polyelectrolyte polyacrylate-based hydrogel-SPA) and antipolyelectrolyte–sulfobetaine-based gel with methacrylate polymeric backbone-SBE) up to 581 J kg^−1^ and a mean power of 0.16 W kg^−1^ was obtained by the tandem setup of SPA and SBE hydrogel containing 3% crosslinking density and particle size of 500 microns with an external load of 3.0 kPa. Exchange of sulfobetaine with methacrylamide (SBAm), the main polymer backbone, revealed a positive increase in energy recovery of 670 J kg^−1^ with a mean power of 0.19 W kg^−1^ for the tandem system operating under the same parameters (SPA@SBAm). The energy recovery can be controlled, modulated and tuned by selecting both hydrogels with antipolyelectrolyte and polyelectrolyte effects and their performing parameters. This proof of concept provides blue energy harvesting by contributing both polyelectrolyte and antipolyelectrolyte effects in a single tandem setup; together with easy accessibility (diaper-based materials (SPA)) and known antibiofouling, these properties offer a robust alternative for energy harvesting.

## 1. Introduction

Continual depletion and increasing consumption of fossil fuel as well as the subsequent boosting of greenhouse emissions have made energy security and climate change top global challenges [1,2]. Alternative energy production from renewable and accessible resources is thus in high demand by society. Moreover, the research community has been strengthened and empowered to investigate more efficient alternatives to conventional approaches. Since seawater is accessible and the most abundant natural resource, it is highly attractive for its study and research on new approaches of energy production. The most investigated extraction and production of energy approaches are based on so-called “blue energy” that results from salinity gradients. Pressure-retarded osmosis (PRO) [3,4,5,6] and reverse electro dialysis (RED) [7,8,9,10,11] are membrane approaches, and their drawback is biofouling, limited resistance and durability of membranes [12]. Biofouling, sophisticated setup and charge leakage are also limitations for membrane-free electrochemical “CAPMIX” [13,14,15,16,17,18,19,20,21,22,23,24] approaches, such as capacitive double layer expansion (CDLE) [18,19], the capacitive Donnan potential (CDP) [20,25], the battery-based MEB technologies (pseudo-capacitive battery reaction or entropy battery) [26] and vapor pressure compression capacitance [27]. Concentration flow cells (CFCs) are the another emerging method for the salinity gradient energy harvesting, which combines the responsive mechanisms for the production of power [28] based on both RED (Donnan potential) and CAPMIX (electrode potential). The MnOx/biochar composite is reported to be an efficient material showing high performance of low electrode resistance and high specific capacitance, which provides efficient salinity gradient energy recovery in CFCs with low cost and good stability [29].

Apart from such approaches, the translation of chemical energy from salinity gradient into mechanical energy by a hydrogel-based osmotic engine [30,31] is another accessible approach for energy conversion. In a single osmotic engine, the power is produced by differences in the swelling ratio of hydrogel matrix in various salinity gradients leading either to swelling of hydrogel and moving a piston with a particular load upward or shrinking and moving the piston with the load downward. Such process can be repeatable in the cyclic mode similar as in a combustion engine. Two complementary polyelectrolyte and antipolyelectrolyte effects were used for energy extraction from salinity gradients. An osmotic engine driven by a polyelectrolyte effect possesses higher expansion of hydrogel matrix in desalinated water and moves a piston upward, while lower expansion in seawater moves the piston downward [32]. Most of studies on such an osmotic engine are poly(acrylic acid) (PAA)-based hydrogel that are superabsorbent and commonly used for children’s diapers. It was observed that hydrogel could generate a maximum mean power up to 0.23 W kg^−1^ calculated to dry form [31] modulated by hydrogel architecture or polymer composition up to an external load of 6 kPa, a polymer with 1.7 mol % crosslinking, a degree of neutralization of 10 mol % and a particle size of 370–670 µm. Additionally, poly(acrylic acid)-based copolymer hydrogels with semi-interpenetrating network exhibiting a higher swelling rate could recover even higher energy when compared to that of PAA hydrogel itself [33]. Apart from the energy recovery from salinity gradients, by applying external weights, Bui et al. demonstrated an osmotic engine with mechanical energy transmission prototype, which converts energy from salinity gradient to a hydraulic accumulator by utilizing copolymer hydrogel with polyelectrolyte effect [34]. In contrast, we have studied the engine principle where the driven force is complementary and opposite to the salinity gradient with a so-called antipolyelectrolyte effect. In this principle, hydrogel matrix swelling in response to seawater and movement of pistons is upward, and subsequent deswelling is in response to desalinated water with a downward movement of pistons. Polyzwitterionic sulfobetaine-based hydrogel use and maximum mean power generated from salinity gradient reach up to 28.6 mW kg^−1^ (calculated based on dry form), obtained by a hydrogel with a 3% crosslinking density, a 200–300 μm particle size, and 100 g as an external load [35]. Energy efficiency in this combination was high at 0.73%. It should be mentioned that in all mentioned approaches, energy is generated only by decreasing or increasing the salinity gradient.

In this contribution, we describe an osmotic engine device containing two compartments with pistons in tandem configuration that possess antipolyelectrolyte- and polyelectrolyte effect-based hydrogels. The advantages and novelty in this principle is that such device enables energy production in both modes, decreasing and increasing the salinity gradient and creates an accessible and cost-effective hydrogel particle source. Two types of antipolyelectrolyte-based hydrogel particles (sulfobetaine-based hydrogel differed in type of main polymeric backbone, namely, polymethacrylate (SBE) and polymethacrylamide (SBAm)) and two types of polyelectrolyte hydrogel particles (polyacrylate-based hydrogel (SPA) as a simple diaper and synthetic polyacrylic acid) were chosen for the hydrogel-based matrices of an osmotic engine. Tandem setup hydrogel particle modification as a matrix and load for piston, in the engine together with repeatability of cycles, were examined.

## 2. Results and Discussion

### 2.1. Design of Study

**Figure 1:**
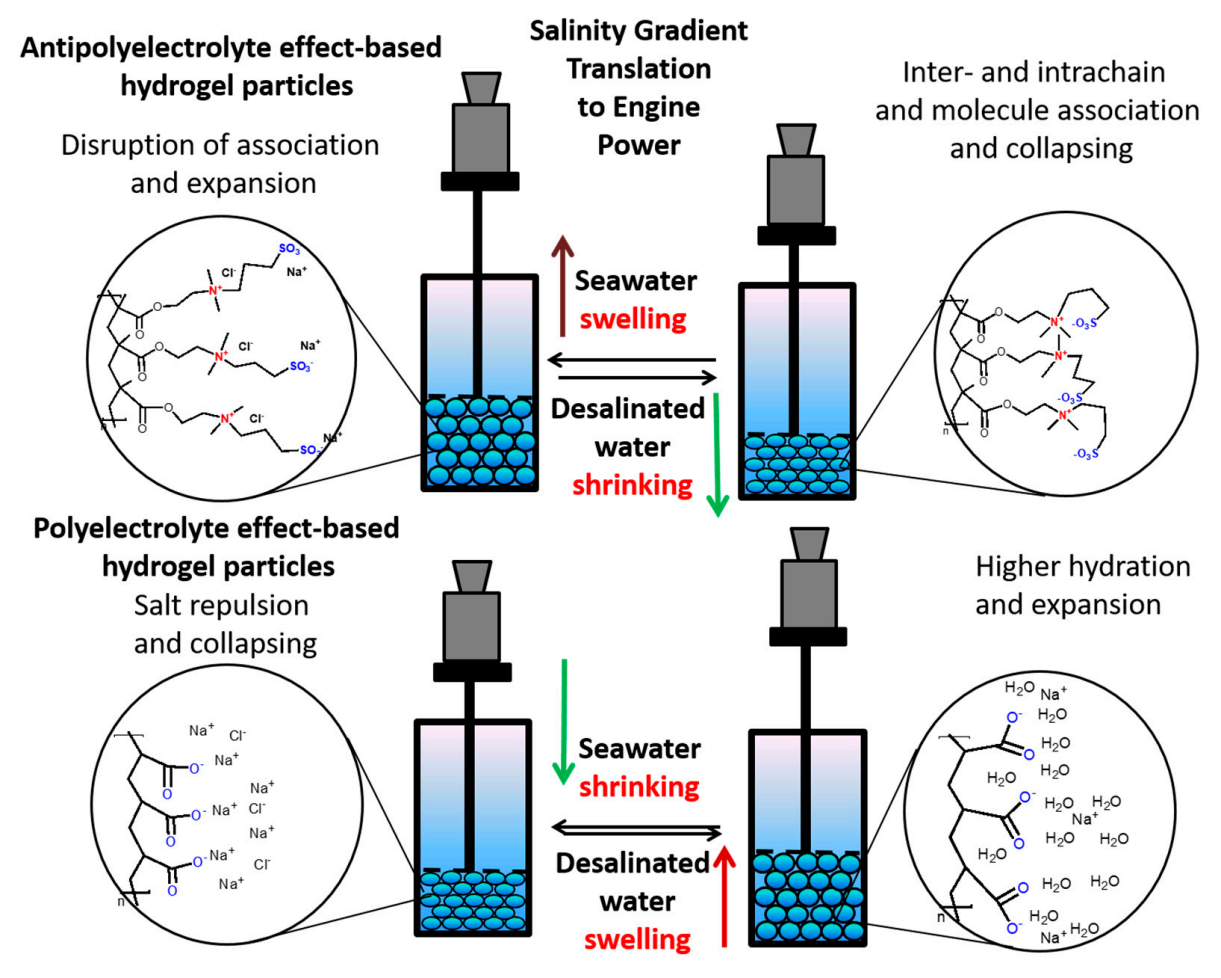
Representation of salinity gradient engine in a tandem setup by combining both hydrogel particles possessing polyelectrolyte and antipolyelectrolyte effects.

The purpose of this study is to evaluate the performance of an osmotic engine assembled with pistons containing hydrogel particles with antipolyelectrolyte and polyelectrolyte units in a tandem arrangement. The swelling of hydrogel particles with a polyelectrolyte effect and the shrinking of hydrogel particles possessing an antipolyelectrolyte effect in desalinated water as well as the shrinking of hydrogel particles with a polyelectrolyte and swelling of hydrogel particles with antipolyelectrolyte effect in saline water generate power in both increasing and decreasing salinity modes (Figure 1). In such setup, it is required to understand proper connection as well as investigate simple and affordable hydrogel alternatives.

**Scheme 1:**
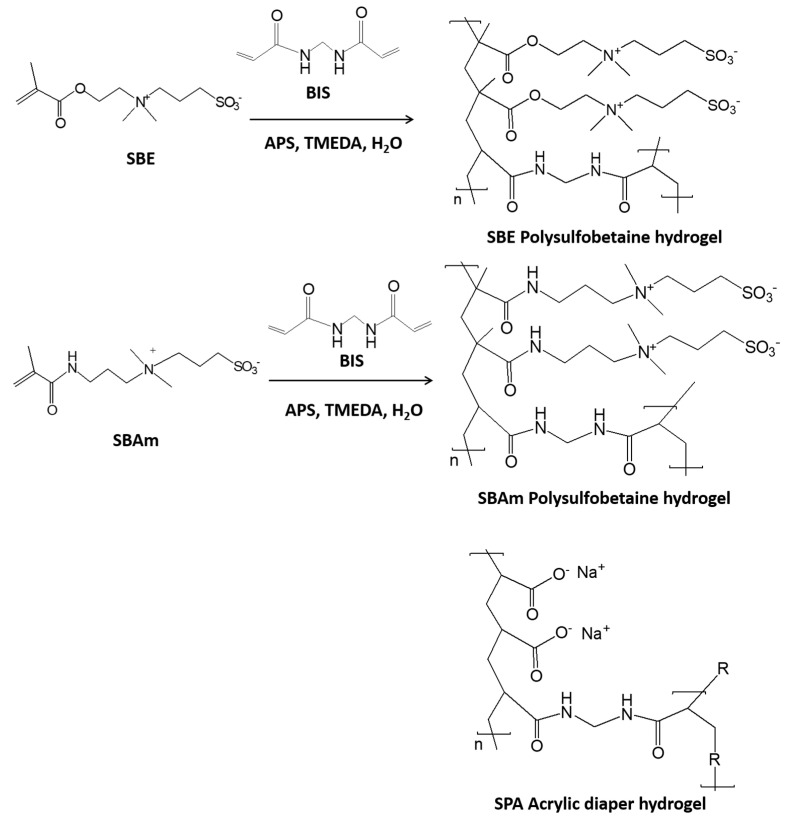
Schematic presentation of SBE, SBAm and SPA hydrogel and synthesis of SBE and SBAm hydrogel.

For this purpose, polyzwitterionic hydrogels with ester moiety as an oxy ethyl functional group in the main polymeric chain (SBE hydrogel of different crosslinking densities, 1.5 mol % and 3.0 mol % of BIS to sulfobetaine monomer (Monomer:BIS) were prepared and denoted as SBE-1.5 and SBE-3.0, respectively) and with amidic (SBAm hydrogel with 3.0 mol % of BIS to sulfobetaine monomer is denoted as SBAm-3.0) were synthesized in the lab and obtained as hydrogel with antipolyelectrolyte effect (Scheme 1). Notably, SBE was also scrutinized due to much higher cost effectiveness and better availability [36] compared to SBAm amide congener [37]. Sodium polyacrylate (SPA) hydrogel extracted from a commercial diaper (Pampers) was considered as the hydrogel with polyelectrolyte effect (Scheme 1). Use of SPA also opens up new avenues to upcycle expired diaper gels in energy generation applications [38]. Tandem arrangements offer energy gains in both modes of salinity gradient, and this smart setup can be further applied in underwater robotics systems and engineering.

### 2.2. Characterization

Crosslinking density has direct influence on the degree of swelling. The degree of swelling is a useful parameter indicating the relative expansion of the hydrogel network in aqueous media with different salinities, thereby defining the length of piston movement and the energy generated by the salinity gradient engine. Dynamic scattering (DS) is different for different materials (SBE and SBAm) and varies for the same material with different crosslinking densities (SBE-1.5 and SBE-3.0), as can be seen from the values provided in Table 1. SBE hydrogels with different crosslinking densities were fabricated by free-radical crosslinking polymerization, and their resultant swelling ratios were calculated. Among SBEs, the hydrogel with crosslinking density 1.5 mol % (with respect to BIS to sulfobetaine monomer ratio (SBE-1.5) exhibits a higher degree of swelling (13.2 g g^−1^) in simulated saline water, with varying values by following the order SBE-1.5 > SBE-3.0. The degree of swelling in DI water follows a similar trend to that in saline water. This observation is different from the pattern observed with sulfobetaine based hydrogel with amidic functionality. Based on our previous observation, sulfobetaine based hydrogel with amidic functionality (11.7 g g^−1^) showed a higher swelling ratio in saline water compared to the one prepared in the current study with hydroxyl functionality (SBE-3.0) (8.4 g g^−1^), at a similar crosslinking density of 3.0% [35]. The lower value of degree of swelling of SBE in 3.6% NaCl can be attributed to the intra- and interpolymeric hydrogen bonding between the oxygen atom on the sulfobetaine chain and water molecules. Moreover, a characteristic of the main polymer chain SBE is ester and for SBAm is amide from methacrylic acid. The amide group of SBAm can interact with lone electron pairs and create additional hydrogen bonding from N–H; this has been confirmed by several studies, including the EWC DSC study as well as a previous study by NMR on SBA and SBE-based polymers [39]. Moreover, the aforesaid hydrogen bonding ability also limits uptake of salt molecules into the SBE hydrogel network compared to more volume of the gel network existing in the sulfobetaine gel with amidic functionality (SBAm) [40]. It conversely affects the deswelling process as well, since the hydrogen bonding holds back the hydrogel from its maximum shrinking in desalinated water, where it retains a higher amount of water. SBE gel shrinks less than SBAm, as observed by their difference in degree of swelling values at 5.2 g g^−1^ and 3.7 g g^−1^, respectively. The intrapolymeric hydrogen bonding in the hydrogel acts as a limiting factor for uptaking the saline water to a level and blocks the hydrogel from its maximum swelling.

**Figure 2:**
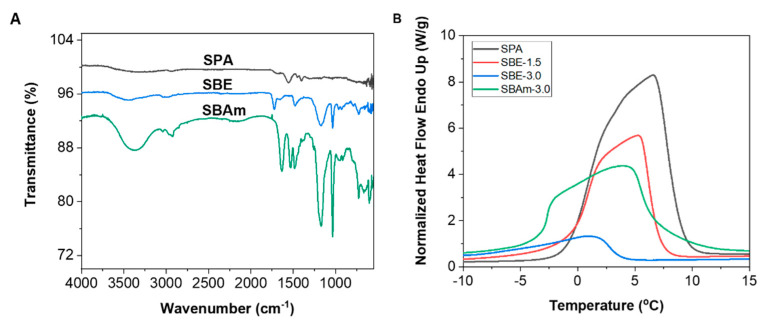

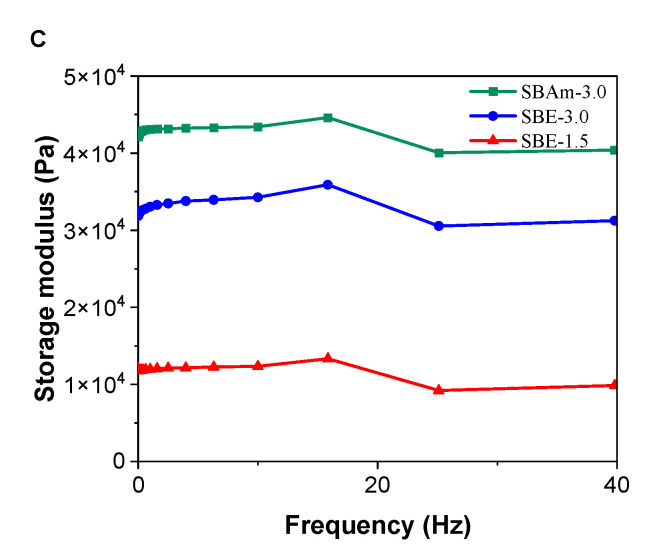
(**A**) FTIR; (**B**) DSC spectra of SBE, SBAm and SPA hydrogel materials; (**C**) Frequency sweep for storage modulus of SBE and SBAm hydrogel samples.

The hydrogel materials are evaluated by the Fourier transformed infrared spectroscopy (FTIR) technique (Figure 2A). FTIR peaks are related to different motions taking place in chemical bonds. SPA is the sodium salt of poly(acrylic acid); hence, this affirms that the existence of bands at 1560 and 1404 cm^−1^ are ascribed to carboxyl group stretching of acrylate. SBE has characteristic absorption bands at 1724, 1484, 1169 and 1033 cm^−1^ that are attributed to a stretching vibration of C=O ester carbonyl group, N-CH_3_ scissoring, symmetric SO_3_ stretching vibration and stretching overlapped with N–C vibration, respectively. SBAm sample showed typical absorption peaks for amide for C=O stretching vibration group at 1635 cm^−1^, secondary amide N–H bending at 1532 cm^−1^ and N–H stretching at 3370 cm^−1^. S–O stretching and C–N vibration were observed at 1168 and 1032 cm^−1^, respectively. 

Water structure of the synthesized hydrogel materials, along with commercial SPA hydrogels, was assessed using the DSC technique with a combination weight of lyophilized samples to determine EWC (summarized in Table 2). It is well known that the water structure in the hydrogel structure network can be divided into different states as freezable water and nonfreezing bound water [41,42,43]. Equilibrium water content can be obtained from lyophilized water, and such value indicates total water contents. The water state assigned as total freezable water (W_tf_) contains free (W_f_) and freezable bound water (W_fb_) that has no or weak interaction with the polymeric network and has a slight influence on transition temperature. Such type of water structure can be obtained from dynamic scattering calorimetry (DSC) by integration of the area under the endothermic curve for water-swollen hydrogels based on Equation (1).

(1) $$W_{\mathrm{tf}}=W_{f}+W_{\mathrm{fb}}=\frac{\Delta H\times 100}{\Delta \mathrm{Hw}}$$

where ΔH and ΔHw are measured enthalpy of sample from DSC measurement and bulk water ΔH_w_ = 333 J g^−1^). Finally, nonfreezing bound water (W_nf_) is structured with the polymer network through strong hydrogen bonds and can be estimated from Equation (2).

W_nf_ = EWC − W_tf_ (2)

DSC endothermic curves of hydrogels are depicted in Figure 2B, and all maxima are shifted to temperatures above 0 values due to the presence of total freezing and nonfreezing water in the polymeric network. Such shift is typical for hydrogel with high affinity to hydrogen bonding [41,42,43]. The highest value for EWC, as well as freezable water, is in SPA, and this is attributed to the superabsorbent ability of sodium polyacrylate-based materials used in hygienic and agricultural application. Moreover, water molecules also interact with sodium ions’ hydration. Additional aliphatic spacers between ions in zwitterionic reduce interaction with water and the degree of freezable water part in zwitterionic-based materials. The amidic polymer backbone function possessing more water than ester SBE-based hydrogels due to additional hydrogen bonding from N–H is a secondary amide group.

Additionally, viscoelastic properties of hydrogel samples were examined (Figure 2C). Frequency sweep was performed at ambient temperature and showed that the SBA3 hydrogel sample was hydrogel with the highest stiffness with a storage modulus at 42 kPa. On the other hand, a much lower storage modulus of 12 kPa was found for SBE 1.5 hydrogel sample. It is worth mentioning that values for EWC have been shown to be slightly higher compared to SBE-based hydrogel prepared with higher concentration for monomer in feed [41].

### 2.3. Performance of Combined Salinity Gradient Engine

As mentioned, this study evaluates the performance of two osmotic engine units in tandem operation, one with polyelectrolyte hydrogel and the other filled with antipolyelectrolyte. The operation of the entire system will particularly depend on the salinity level of the solution entering system and the point of solution entrance, whether it is initially entering the polyelectrolyte system followed by the antipolyelectrolyte or vice versa.

For instance, when saline water is introduced to the antipolyelectrolyte hydrogel, it swells and results in the upward piston movement, while introducing desalinated water moves the piston downward, accompanying shrinkage in SBE hydrogel. The polyelectrolyte behaviour of SPA makes it swell in desalinated water, while shrinking in saline water moves the piston upward. Mechanical work performed by the piston during upward and downward movement with different external loads attached to the piston leads to mechanical energy production.

Two different tandem assemblies are evaluated to understand the influence caused by the connection and which connection renders faster piston movement and higher energy generation along with higher energy efficiency.

Connection A: Introduces the seawater inlet to the first compartment from the cylinder with the piston containing antipolyelectrolyte hydrogel particles followed by subsequent entering solution to the second compartment with the piston filled with polyelectrolyte hydrogel particles. Both SBE and SPA hydrogels in an equilibrium swollen state in DI water were used to pack the engines.

Connection B: Introduces the seawater inlet to the cylinder containing polyelectrolyte, followed by subsequently entering the second engine filled with antipolyelectrolyte hydrogel particle. Similar to connection A, the engine was packed with hydrogels in their respective equilibrium swollen states in DI water, and in both tandem setups, water inlet was connected to the seawater container and subsequently switched to the DI water container.

**Figure 3:**
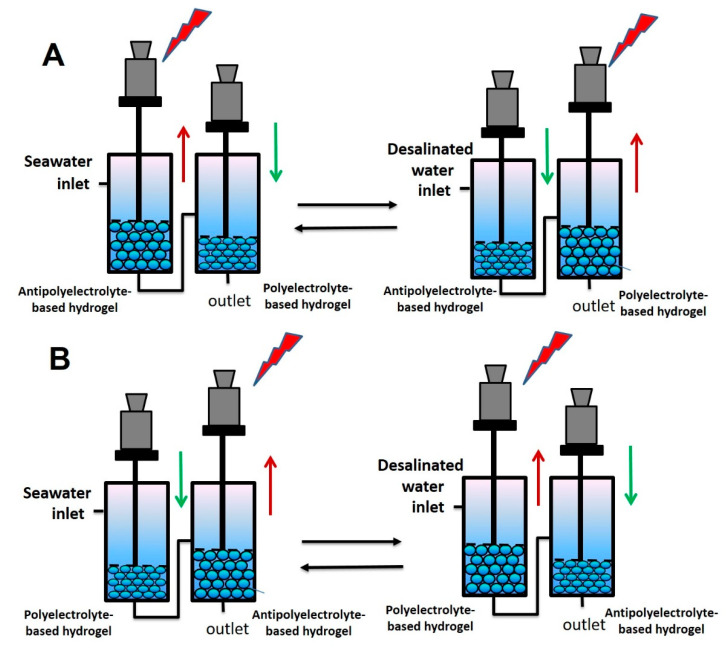
Schematic representation of solvent entrance and related hydrogel movement in different operating modes, (**A**) connection A, (**B**) connection B.

In connection A, the solvent entry point is at the antipolyelectrolyte SBE hydrogel. At the start, SBE is in its equilibrium, deswollen state. Hence, with the introduction of saline water (3.6 w/w % of NaCl), SBE started to swell moving up the piston. While SBE gel was progressing in the swelling behaviour, there was no outflow of the saline water. This resulted in SPA gel remaining stable at its original packed height. After SBE reached a plateau, saline water entering through system 1 reached system 2. This caused SPA to deswell and move the piston down. When the deswelling was complete, indicated by constant low level in SPA over time, the solution was exchanged to DI water. Introducing DI water triggered the swelling process of SPA. During the swelling of SPA, the hydrogel particles were encapsulated with deionized water by releasing salt particles and left system 2 as effluent, entering system 1 equipped with SBE hydrogel. Assessing the quality of the solution entering at system 2, saline in nature due to the salt particles, it withdrew from system 2, and therefore, deswelling of SBE was not as fast as in DI water. Deswelling took place at a lower rate than that with DI water. This is reasonable since the salinity level of the solution lies in between the 3.6 w/w % NaCl solution and DI water. The two processes, namely deswelling of SBE and swelling of SPA, proceeded simultaneously. However, a slow swelling rate was observed with excessive time consumed for the SPA hydrogel to reach the cycle height at start. This is attributed to the lower salinity gradient the system was experiencing with the two solutions exchanged at solution feed point in system 1 (3.6 w/w % NaCl and DI water), even though it is not apparent in the graph (Figure 3A) as a result of the lengthy time scale.

**Figure 4:**
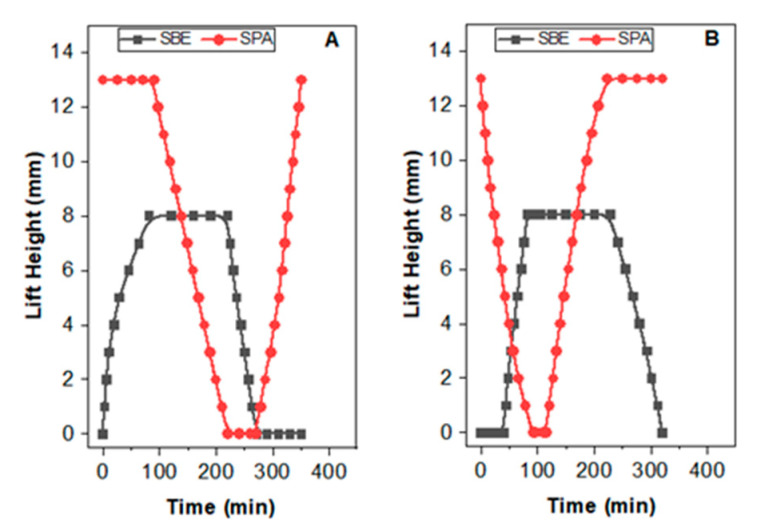
Performance of one cycle of (**A**) SBE–SPA hydrogel engine by introducing saline water to antipolyelectrolyte SBE followed by the desalinated water exchange. (**B**) Performance of one cycle of SBE–SPA hydrogel engine by introducing saline water to polyelectrolyte SPA followed by the desalinated water exchange. In the two cases, applied load was 2.1 kPa. SPA hydrogel used in its commercially available form and SBE-3.0 hydrogel with particle size 700 μm. The flow rate was maintained at a rate of 1 mL h^−1^.

In connection (B), the polyelectrolyte in its swollen state started deswelling by the introduction of saline water. From the performance diagram (Figure 4B), a fast solvent release was observed during the shrinking of polyelectrolyte hydrogel, which facilitated the early swelling of the antipolyelectrolyte (after 40 min from the starting time of SPA deswelling). Even if connection B provided easy solvent release from the first cylinder to the second one, the salinity level of the solution entering at system 2 was lower than that of pure saline water, and the rate of upward movement of piston was found to be subsequently lower than that with 3.6 wt % saline water. The total energy recovered from the engine having connection B was found to be 274.2 J kg^−1^, and that of connection A was found to be 275.9 J kg^−1^ (Table 3). By considering the comparable energy recovery of both system and uninterrupted tracking of the rate of movement of system A during seawater and DI water exchange, the investigation proceeded by connection A.

### 2.4. Effect of Crosslinking Density on Energy Recovery

The swelling ratio is not the only requirement for the hydrogel to act as a good candidate for a salinity gradient engine; it should also possess the mechanical strength to lift the weight upward and downward during swelling and deswelling, respectively. The ability of a hydrogel in power production is unpredictable with respect to swelling ratios. Even though the weakly dense hydrogel provides a higher degree of swelling, it is mechanically weak to lift a higher load and resulted in comparatively lower values. To examine the effect of load on power generation, different external loads of 1.2 kPa, 2.1 kPa and 3.0 kPa were applied to the piston. As the hydrogels of different crosslinking densities do not behave linearly towards different loads, the effects of applied loads on the pistons of cylinders packed with hydrogels of different CDs were examined and compared. The lifting heights of the prepared sulfobetaine-based hydrogels (SBE) of different crosslinking densities by applying different external loads were plotted as a function of time.

Combining these hydrogels (SBE-1.5 and SBE-3.0) separately with the same polyelectrolyte SPA (SPA–SBE-1.5 and SPA–SBE-3.0) by applying the load of 1.2 kPa generated a mean power of 55.3 mW kg^−1^ (with a recovered energy of 200.2 J kg^−1^) and 55.9 mW kg^−1^ (recovered energy of 201.3 J kg^−1^), respectively, with an energy efficiency of 0.06% and 0.08% (Table 4). The lift height of the hydrogel samples towards lower external weights shows an inverse proportion to the crosslinking density. This is because of the difference in swelling ratios of the hydrogel materials. The hydrogel SBE with crosslinking density of 1.5 mol % showed the degree of swelling 13.2 g g^−1^ and provided a faster swelling by uptaking a larger amount of saline water in the loosely crosslinked polymer network, whereas the hydrogel SBE with crosslinking density of 3 mol % showed a degree of swelling of 8.4 g g^−1^ and the hydrogel network as being denser than that of the former. Even if the low weight of 1.2 kPa did not make any impact on the hydrogel particles, the energy recovery and the efficiency were defined by the time taken for the hydrogel material to attain the equilibrium point.

When the external load increased to 2.1 kPa, SBE-1.5 and SBE-3.0 provided an energy recovery of 296.9 J kg^−1^ with an efficiency of 0.24% and 275.9 J kg^−1^ with an efficiency of 0.11%, respectively, from the combined osmotic engine. The external load of 3.0 kPa, attained by SBE-1.5 in 179 min, produced an energy recovery of 577.0 J kg^−1^ with an efficiency of 0.19%, and in 143 min SBE-3.0 produced a total energy of recovery of 571.2 J kg^−1^ with an efficiency of 0.08%. In the case of SBE-1.5, the load increased from 1.2 kPa to 3.0 kPa in an inversely proportional relation. This is attributed to the weak resistance of the least dense hydrogel towards the higher weights. However, the rate of movement of the piston to reach the equilibrium level has also been found to be decreasing with increasing the external load on the piston. In all cases, increasing the load increased the mean power generated and energy recovery irrespective of the crosslinking density.

### 2.5. Effect of Zwitterionic Polymeric Backbone

**Figure 5:**
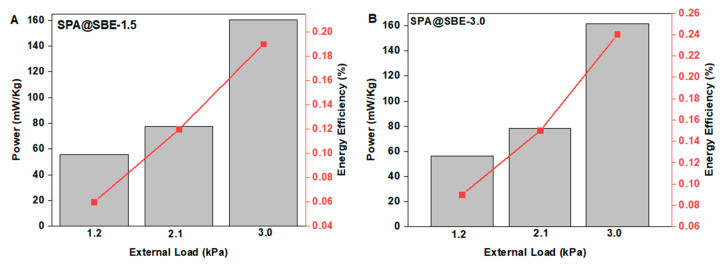
Power generated by combined osmotic engine with SPA and SBE of crosslinking densities 1.5% and 3.0% with the particle of 700 micron. The flow rate maintained as 1 mL h^−1^.

To investigate the effect of crosslinking density on energy recovery, the combined osmotic engine with SPA as polyelectrolyte and sulfobetaine hydrogel SBE with two different crosslinking densities of 1.5 mol % and 3.0 mol % were chosen as antipolyelectrolyte and subjected for the experiment. An optimal load of 2.1 kPa was considered to check the effect of the density of hydrogel on power generation and energy recovery. Increasing the density of hydrogel from 1.5% to 3.0%, the total mean power was found to be decreasing from 1.76 to 1.69, but considering power recovery per kilograms of dry hydrogel mass, the combined salinity gradient engine with SBE-1.5 produced a total power of 77.4 mW kg^−1^ and an energy of 278.2 J kg^−1^, which was higher than that with SBE-3.0 (power of 76.8 mW kg^−1^ and energy of 275.9 J kg^−1^) (Entries 5 and 8 in Table 4). The calculated energy efficiency of the combined osmotic engine with SBE-3.0 as antipolyelectrolyte was 0.11%, and that with SBE-1.5 was 0.12%. The total power generated and corresponding energy efficiencies by the osmotic engines SPA@SBE-1.5 (Figure 5A) and SPA@SBE-3.0 (Figure 5B) by the external loads of 1.2 kPa, 2.1 kPa and 3.0 kPa respectively are shown in Figure 5. Apart from the performance of sulfobetaine hydrogel with amidic functionality (SBAm) of 3% crosslinking [35], the sulfobetaine hydrogel containing the oxy ethyl group (SBE) exhibited a slightly better performance with a crosslinking density of 1.5% than that with 3.0% towards an external load of 2.1 kPa in the energy recovery. Proper spacing in chains and chain fragments provides better water diffusion and thereby faster swelling and shrinking, which facilitate higher power and energy recovery. To achieve proper spacing between the fragments, the crosslinking ratio (crosslinker to monomer) should not be higher. In the case of SBE, the contradictory result of lower power achieved by the crosslinking density of 3% when compared to that of 1.5% might be because of the presence of hydrogen bonding between the oxygen atoms on the monomer units to the hydrogen atoms of the crosslinker moieties, which inhibits the faster swelling and deswelling of hydrogel. The spacing between the fragments should be higher in the case of hydrogels with CD of 1.5% and thereby lower the effect of hydrogen bonding, which facilitate higher energy recovery.

### 2.6. Performance of SBE@SPA Hydrogel Engine towards Different Loads

**Figure 6:**
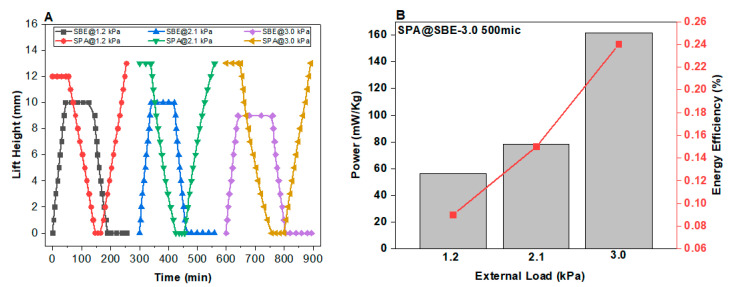
(**A**) Performance of one cycle of SBE–SPA hydrogel particle engine by applying different external loads during seawater and desalinated water exchange exhibiting equilibrium. SPA hydrogel used in its commercially available form and SBE hydrogel was with CD-3.0 and particle size of 500 μm. The flow rate was maintained at a rate of 1 mL h^−1^. (**B**) Power generated by the engine along with the energy efficiency.

The movement of the piston along the upward and downward direction and thereby the power generation is affected by the external weight attached to the piston of an osmotic engine. The loads of 1.2 kPa, 2.1 kPa and 3.0 kPa were applied to check the effect of external weight on energy recovery and efficiency (Figure 6A). In the combined engine setup, the syringes each containing both polyelectrolyte and antipolyelectrolyte were loaded with the same load to investigate the impact of external weight. By introducing saline water, the antipolyelectrolyte swells and reaches its point of equilibrium. At the plateau, the saline water was replaced with desalinated water, and deswelling was initiated. The power produced is calculated based on cycle time. The exact reverse process was carried out in the polyelectrolyte. The addition of the power generated in each compartment provides the total mean power of the combined osmotic engine. The behaviour of both polyelectrolyte and antipolyelectrolyte towards the external loads has been previously studied as a single system [31,35]. The performance of hydrogels towards the external weights depends on the swelling ability, density of hydrogels and the nature of functional group in the polymer back bone. Any functional group that facilitates the hydrogen bonding with deionized water may lead to the presence of nonfreezable bound water inside the hydrogel network and affect the swelling ratios. Thus, the performance of each hydrogel towards the external weight may vary according to their ability to lift the corresponding load during swelling and the pressure applied on the hydrogel by the external weight attached to the piston.

With a load of 1.2 kPa, the pat-dried, deswollen mass of the sulfobetaine hydrogel SBE with cross linking density of 3 mol % and the particle size of 500 μm started swelling during the introduction of saline water, and the swollen, pat-dried mass of polyelectrolyte (SPA) started deswelling, and the piston was moved to a point of their own equilibrium. When the saline water was replaced with desalinated water, the antipolyelectrolyte started deswelling, and the polyelectrolyte started swelling to their point of equilibrium. The impact of the external load 1.2 kPa on the combined engine generated a power of 56.3 mW kg^−1^ and a total energy of 202.9 J kg^−1^ with an energy efficiency of 0.09% (Table 4). The combined engine with load of 2.1 kPa generated a mean power of 78.3 mW kg^−1^ and an energy of 281.1 J kg^−1^. The energy efficiency of the engine with a load of 2.1 kPa was found to be 0.15%, which is higher than the engine with an applied load of 1.2 kPa. By applying a higher load of 3.0 kPa, the ability of antipolyelectrolyte to lift the load upward was found to be weak by providing a translational movement, which is lower than that with low applied loads. The combined engine with an external load of 3.0 kPa generated a total mean power of 161.6 mW kg^−1^ and a total energy of 581.1 J kg^−1^ with a maximum efficiency of 0.24%.

### 2.7. Energy Recovery from the Osmotic Engines of Two Different Assemblies

For investigating energy recovery, two types of sulfobetaine hydrogels, one having oxy ethyl group (SBE) and other having amidic functionality (SBAm), were chosen as antipolyelectrolyte and SPA as polyelectrolyte in fabricating the combined osmotic engine. The effect of particle size, crosslinking density and external load were investigated by combining the SBE hydrogel with polyelectrolyte hydrogel SPA; their power generation and energy recovery is tabulated. The sulfobetaine hydrogel SBAm and its effects of particle size, crosslinking density and the external load in energy recovery were reported recently by our group [35]. Thus, the SBAm hydrogel with reported maximum efficiency (density of 3%) was chosen for comparing the energy recovery from the sulfobetaine hydrogel with the oxy ethyl group (SBE) by keeping the particle size as 500 microns. The recovered energy values are shown in Table 5.

**Figure 7:**
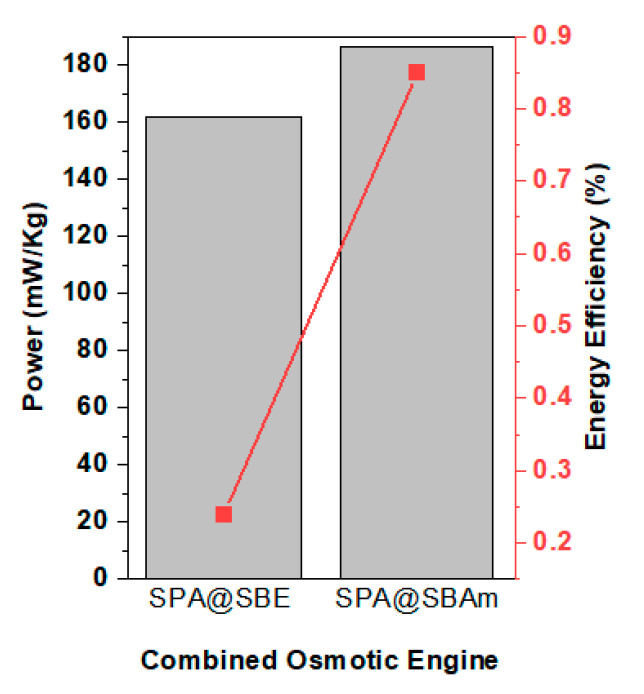
Power recovered by three different combinations of polyelectrolytes and antipolyelectrolytes with crosslinking density of 3.0 mol % and particle size of 500 μm with a load of 3.0 kPa. The flow rate was maintained as 1 mL h^−1^.

The energy recovery from combined osmotic engines having both polyelectrolyte and antipolyelectrolytes were investigated by choosing the hydrogels with their most efficient compositions. In the present study, the prepared sulfobetaine hydrogel (SBE) provided the maximum power generation and energy recovery with crosslinking density 3.0% and particle size of 500 μm with an external load of 3.0 kPa as the combined engine with polyelectrolyte SPA as its pair. The energy recovery from the engine per kilograms of dry hydrogel mass was found to be 581.1 J kg^−1^ with an efficiency of 0.24%. The osmotic engine when antipolyelectrolyte SBE was replaced by SBAm provided energy of 670.0 J kg^−1^ with an efficiency of 0.85% (Figure 7). The chemical structure of SBE and SBAm are similar, except for the functional group in which SBE has the oxy ethyl group in the main chain and SBAm has amidic functionality instead.

### 2.8. Reusability of the Polyelectrolyte and Antipolyelectrolyte over Cycles

**Figure 8:**
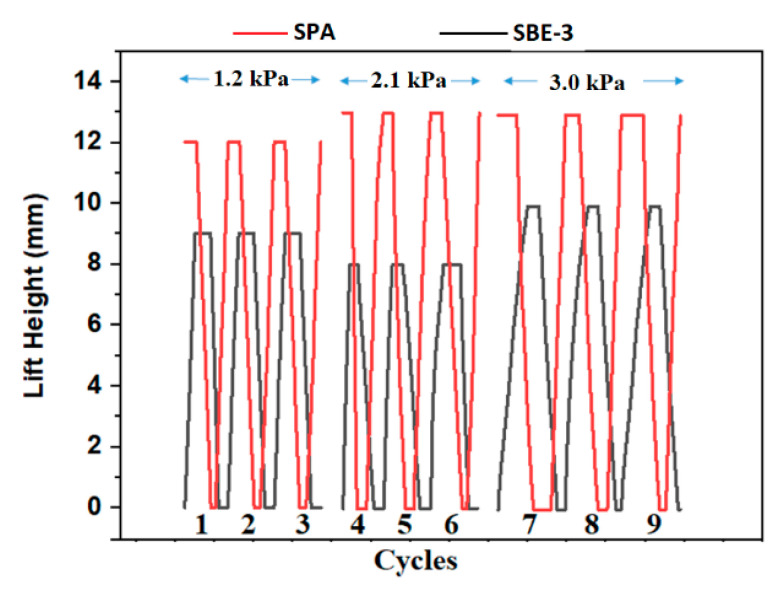
Performance of sulfobetaine–sodium polyacrylate hydrogel engine by applying different external loads during seawater and desalinated water exchange exhibiting equilibrium. SPA hydrogel was used in its commercially available form, and SBE hydrogel was with CD-3.0 and particle size of 700 μm. The flow rate was maintained at a rate of 1 mL h^−1^.

The reusability of hydrogels was investigated by analysing the performance of sulfobetaine–sodium polyacrylate hydrogel engine by applying different external loads during seawater and desalinated water exchange over nine cycles (Figure 8). With applied loads of 1.2 kPa, 2.1 kPa and 3.0 kPa, both the antipolyelectrolyte and polyelectrolyte hydrogels performed accordingly well by keeping the repeatability of performance over nine cycles. The SPA hydrogel from commercially available diaper and sulfobetaine hydrogels of crosslinking density 3.0 and particle size of 700 μm were chosen by checking the repeatability of the combined osmotic engine. Both hydrogels behaved exclusively on their own weightlifting capacity and swelling characteristic towards each external load by keeping the consistency in each three cycles with the same external load for a total of nine cycles. The antipolyelectrolyte SBE-3 and the polyelectrolyte SPA maintained the consistency over three cycles with the same external loads. The rate of movement of both hydrogels with a load of 3.0 kPa is found to be lower than that with a load of 2.1 kPa. The power generated by all these combined osmotic engines with different external loads are shown in Table 2.

## 3. Materials and Methods

### 3.1. Materials

*N,N′*-Methylenebis(acrylamide) (BIS), *N,N,N′,N′*-tetramethylethylenediamine (TMEDA), ammonium peroxodisulfate (APS), [2-(methacryloyloxy) ethyl] dimethyl-3-sulfopropyl) ammonium hydroxide (SBE) and *N*-(methacryloylaminopropyl)-*N*,*N*-dimethyl-*N*-(3-sulfopropyl) ammonium betaine (SBAm) were purchased from Sigma–Aldrich in the highest available grade and used without further purification. Sodium polyacrylate hydrogels (SPA) were separated from the commercially available diaper (Pampers) and taken as the polyelectrolyte hydrogel. For all preparations, Milli-Q deionized (DI) H_2_O from a Millipore setup was used and modelled as desalinated water. As an artificial seawater-like solution, 3.6% w/v solution of NaCl was prepared.

### 3.2. Hydrogel Particles Sample Preparation

A total of 2.27 g (10 mmol) of SBE and the appropriate amount of BIS were dissolved in 5 mL of DI water. Then, 400 μL of 0.22 M APS was added, and the solution was degassed by nitrogen for 10 min. Subsequently, 140 μL of TMEDA was added and sealed, and the polymerization process proceeded for 6 h, yielding SBE hydrogel. For purification, the hydrogel formed was immersed in DI water, which was changed several times for three days. The hydrogel samples of different crosslinking densities, 1.5 mol % and 3.0 mol % of BIS to sulfobetaine monomer (Monomer:BIS), were prepared and denoted as SBE-1.5 and SBE-3.0, respectively. For the synthesis of SBAm hydrogel particle, the same procedure was followed with SBAm instead of SBE. The swelling characteristics of the hydrogels are listed in Table 4. The prepared hydrogel samples were crushed and passed through appropriate mesh sizes to obtain hydrogel particles.

### 3.3. Characterization

The DSC was performed by DSC 8500 instrument (Perkin Elmer, USA). Before measurement, the DSC was calibrated with water and indium standards. Hydrogel samples were wiped, cut (5–20 mg), weighed and immediately sealed in Al pan Cooled 20—45 and heated with a heating rate of 10 C min^−1^ under N_2_ flow. All experiments were carried out in duplicate. Fourier transformed infrared spectroscopy with attenuated accessory (FTIR-ATR) was examined with the Spectrum 400 (Perkin Elmer, Waltham, MA, USA). The dynamic mechanical analysis (DMA) testing was carried out by RSA-G2 (TA Instruments, New Castle, DE, USA) at ambient temperature.

Calculation of degree of swelling (DS); degree of swelling in deionized water DS (DI): A gently cut and pat dried sample was weighed (m_DI_). After drying at a temperature of 110 °C for 16 h, it was subsequently weighed (m_D_), and the swelling ratio was calculated using Equation (3) as follows:

(3) $${\mathrm{DS}}_{\left(\mathrm{DI}\right)}=m_{\mathrm{DI}}/m_{D}$$

Degree of swelling in 3.6 wt % NaCl solution DS _(NaCl)_: A gently dried hydrogel sample was weighed (m_NaCl_) and subsequently immersed in 3.6 wt % NaCl solution. To ensure that equilibrium for the degree of swelling was reached, the salt solution was changed several times for 16 h, and the sample was weighed (m_eqNaCl_).

(4) $${\mathrm{DS}}_{\left(\mathrm{NaCl}\right)}={\mathrm{DS}}_{\left(\mathrm{DI}\right)}\times m_{\mathrm{eqNaCl}}/m_{\mathrm{NaCl}}$$

Equilibrium water content (EWC) was calculated using the equilibrium Equation (5)

(5) $$\mathrm{EWC}=\frac{m_{D}-m_{\mathrm{DI}}}{m_{\mathrm{DI}}}$$

### 3.4. Salinity Gradient Engine Setup

Two modified syringes were used for the combined energy recovery engine setup. Cloth wire gauze was inserted in the bottom of each syringe cylinder in order to prevent the hydrogel from passing through the outlet. The solution inlet was the needle tip at a higher position on the syringe barrel, and the outlet was the syringe tip. In this experiment, one syringe was filled with a known quantity of antipolyelectrolyte hydrogel with appropriate particle size and crosslinking density, and the other syringe was filled with a known quantity of polyelectrolyte hydrogel. The two syringes were connected by a flexible tube in a way that the outlet of the first cylinder served as the source for the inlet of the second cylinder to obtain a combined engine setup. The solution-filled syringes, which connected to a syringe pump, acted as the external sources of both the saline and desalinated water and provided a constant rate of flow throughout the swelling and shrinking cycles. The syringe, which was connected directly by the external solution source, was kept at a higher position when compared to the second one for the flawless flow of solution from the first syringe piston to the second piston. All experiments were carried out in duplicate.

### 3.5. Calculation of Energy Recovery and Power Generation

The energy recovery of the osmotic engine was measured by the appropriate equations [35]. As the power generation in a salinity gradient engine is based on the swelling and shrinking of the hydrogels by applying external loads, the mean power provides significant values for calculating energy translated during the swelling and deswelling processes. The mean power is the average power generated over one cycle, in which the time starting from when the swollen antipolyelectrolyte hydrogel reaches equilibrium in DI water to the time when the hydrogel swollen in 3.6% wt/v solution of NaCl nearly reaches equilibrium. The equation for determining the mean power $\bar{P}$ is as follows:

(6) $$\bar{P}=\frac{\Delta W}{\Delta t}=m\times g\times \frac{\Delta h}{\Delta t}\;$$

where ΔW is the work, Δt is the time required for one cycle, m is the mass moved by the piston, g is the gravitational constant of 9.8 m s^−2^ and Δh is the lifting height.

In the salinity gradient engine, as work is performed both by the external load applied on the piston and the swelling of the hydrogel, different parameters such as average hydrogel mass, average change in centre of gravity need to be considered (Equation (7)).

(7) $$\bar{P}=\frac{W1}{\Delta t}+\frac{W2}{\Delta t}=m1g\;\frac{\Delta h_{1}}{\Delta t}+\;m2g\;\frac{\Delta h_{2}}{\Delta t}\;$$

where m1 is the applied load, m2 is the average hydrogel mass in the swollen and shrank state,$\;\Delta h_{1}$ is the average lift height and $\Delta h_{2}$ is the average change in centre of gravity of the hydrogel during swelling and shrinking process.

## 4. Conclusions

The osmotic engine assemblies (namely, SPA@SBE and SPA@SBAm where SBE and SBAm hydrogel particles showing antipolyelectrolyte behaviour and SPA from diaper as particles with polyelectrolyte behaviour) were set up by arranging hydrogel particles with polyelectrolyte and antipolyelectrolyte effects in a tandem manner. Connection, where the inlet of fluid is first introduced to the piston possessing hydrogel particles with antipolyelectrolyte and then polyelectrolyte effects, generates more energy than opposite connection. The energy recovery of 670 J kg^−1^ (calculated based on dry form for each polyelectrolyte and antipolyelectrolyte) was reached by the combined engine in which SBAm hydrogel obtained 3% crosslinking density and particle size of 500 micron with an external load of 3.0 kPa. This polyelectrolyte–antipolyelectrolyte combined system is comparable and generated a total mean power of 0.19 W kg^−1^ with an external load of 3.0 kPa, which is higher than the mean power recovered by poly(acrylic acid) hydrogel-based polyelectrolyte as a single system (0.23 W kg^−1^ with an external load of 6 kPa) [31]. As the poly(acrylic acid)-based hydrogels exhibit higher swelling ratios, they deform easily under pressure in their swollen state, and that leads to a high pressure drop in the system and thereby energy loss. When the deformation hydrogel particle is high, the flowing liquid may need extra energy, which exceeds the energy generated by the system to flow through the hydrogel particles [44]. The higher water storage capacity of diaper gel leads to the deformation of gel particles and thereby a pressure drop in their swollen state by exerting an applied external load, leading to a significant cycle time and relatively low energy recovery. In the energy recovery systems, this drawback may be overcome by selecting poly(acrylic acid)-based copolymer hydrogels instead of PAA itself.

Furthermore, biofouling is one of the most challenging issues in the application of material in natural water-based environments. Several strategies are applied to prevent biofouling [45,46,47,48]; application of suitable hydrogel is one of them. Thus, it is noteworthy that both hydrogel components as polyacrylic [49] as well as zwitterionic-based materials [50,51] are well known for their antibiofouling properties and underwater stability [52], making them attractive as a robust alternative and exhibiting promising potential, in terms of energy recovery, for blue energy harvesting near seawater–fresh water borders and as a water-salinity-driven actuator in underwater engineering and robotics by adopting appropriate hydrogels with polyelectrolyte and antipolyelectrolyte effects.

It should be pointed out that this investigation is still fundamental, and large applicability and profitability may be limited by slowness of ion exchanges and of swelling–deswelling rates.

## Figures and Tables

**Table 1:** Swelling properties of synthesized hydrogels SBA, SBE and SBA (hydrogel from commercially available diaper—Pampers).

Sample Code	CD	BIS (mol %)	DS_DI_ (g g^−1^)	DS_(3.6 wt% NaCl)_ (g g^−1^)	DS_NaCl_/DS_DI_
SBE-1.5	1.5	1.5	6.6	13.2	2.0
SBE-3.0	3.0	3.0	5.2	8.4	1.6
SBAm-3.0	3.0	3.0	3.7	11.7	3.2
SPA	NA	NA	182.0	59.4	0.3

**Table 2:** Equilibrium water content (EWC) and water state for hydrogel samples.

Sample Code	EWC (%)	W_tf_ (%)	W_nf_ (%)
SBE-1.5	84.7	51.6	33.1
SBE-3.0	80.8	45.8	35
SBAm-3.0	82.0	41.6	40.4
SPA	99.4	90.6	8.8

**Table 3:** Total mean power recovered from different combinations of the osmotic engine from hydrogels with polyelectrolytes (SPA) and antipolyelectrolyte (SBE) effect.

Connection	Load (kPa)	$\bar{P}$ (W × 10^−6^)	E, 1 h (J × 10^3^)	$\bar{P}$, 1 kg (mW kg^−1^)	E, 1 h, 1 kg (J kg^−1^)
A-SBE@SPA	2.1	1.72 ± 0.02	6.11 ± 0.05	76.81 ± 0.04	275.89 ± 0.32
B-SPA@SBE	2.1	1.52 ± 0.01	5.33 ± 0.03	65.72 ± 0.02	274.20 ± 0.08

**Table 4:** Total mean power and energy recovered from the osmotic engine when combining both polyelectrolytes and antipolyelectrolyte of varying crosslinking densities and particles sizes.

Connection Setup	Sample Code	Particle Size of Sulfobetaine Hydrogel (µm)	Load (kPa)	$\bar{P}$ (W × 10^−6^)	E, 1 h (J × 10^−3^)	$\bar{P}$, 1 kg (mW kg^−1^)	E, 1 h, 1 kg (J kg^−1^)
A	SPA@SBE-3.0	500	1.2	1.42 ± 0.02	5.13 ± 0.06	56.32 ± 0.07	202.93 ± 0.12
A	SPA@SBE-3.0	500	2.1	2.41 ± 0.06	8.51 ± 0.21	78.33 ± 0.12	281.09 ± 0.44
A	SPA@SBE-3.0	500	3.0	3.74 ± 0.05	13.39 ± 0.17	161.61 ± 0.09	581.14 ± 0.35
A	SPA@SBE-3.0	700	1.2	1.19 ± 0.02	4.43 ± 0.04	55.94 ± 0.02	201.28 ± 0.08
A	SPA@SBE-3.0	700	2.1	1.72 ± 0.02	6.11 ± 0.05	76.81 ± 0.04	275.89 ± 0.32
A	SPA@SBE-3.0	700	3.0	2.48 ± 0.01	8.82 ± 0.03	159.04 ± 0.02	571.24 ± 0.07
A	SPA@SBE-1.5	700	1.2	1.03 ± 0.01	3.62 ± 0.03	55.63 ± 0.02	200.21 ± 0.06
A	SPA@SBE-1.5	700	2.1	1.79 ± 0.06	6.44 ± 0.23	77.52 ± 0.14	278.18 ± 0.49
A	SPA@SBE-1.5	700	3.0	3.04 ± 0.05	10.63 ± 0.09	160.53 ± 0.05	577.03 ± 0.20
A	SPA@SBAm-3.0	500	3.0	13.53 ± 0.15	48.84 ± 0.54	186.31 ± 0.38	670.04 ± 1.37

**Table 5:** Total mean power and energy recovered from the osmotic engine when combining SPA as polyelectrolyte and SBE and SBAm as antipolyelectrolyte hydrogel particles.

Connection	Sample Code	Article Size of Sulfobetaine Hydrogel (µm)	Load (kPa)	$\bar{P}$ (W × 10^−6^)	E, 1 h (J × 10^−3^)	$\bar{P}$, 1 kg (mW kg^−1^)	E, 1 h, 1 kg (J kg^−1^)
A	SPA@SBE-3.0	500	3.0	3.74 ± 0.05	13.39 ± 0.17	161.61 ± 0.09	581.14 ± 0.35
A	SPA@SBAm-3.0	500	3.0	13.53 ± 0.15	48.84 ± 0.54	186.31 ± 0.38	670.04 ± 1.37

## References

[1] Höök M., Tang X. (2013). Depletion of Fossil Fuels and Anthropogenic Climate Change—A Review. Energy Policy.

[2] Shafiee S., Topal E. (2009). When Will Fossil Fuel Reserves Be Diminished?. Energy Policy.

[3] Helfer F., Lemckert C., Anissimov Y.G. (2014). Osmotic Power with Pressure Retarded Osmosis: Theory, Performance and Trends–A Review. J. Membr. Sci..

[4] Achilli A., Childress A.E. (2010). Pressure Retarded Osmosis: From the Vision of Sidney Loeb to the First Prototype Installation—Review. Desalination.

[5] Cheng Z.L., Li X., Chung T.-S. (2018). The Forward Osmosis-Pressure Retarded Osmosis (FO-PRO) Hybrid System: A New Process to Mitigate Membrane Fouling for Sustainable Osmotic Power Generation. J. Membr. Sci..

[6] Nagy E., Hegedüs I., Tow E.W., Lienhard V.J.H. (2018). Effect of Fouling on Performance of Pressure Retarded Osmosis (PRO) and Forward Osmosis (FO). J. Membr. Sci..

[7] He Y., Huang Z., Chen B., Tsutsui M., Shui Miao X., Taniguchi M. (2017). Electrokinetic Analysis of Energy Harvest from Natural Salt Gradients in Nanochannels. Sci. Rep..

[8] Tufa R.A., Pawlowski S., Veerman J., Bouzek K., Fontananova E., di Profio G., Velizarov S., Goulão Crespo J., Nijmeijer K., Curcio E. (2018). Progress and Prospects in Reverse Electrodialysis for Salinity Gradient Energy Conversion and Storage. Appl. Energy.

[9] Jia Z., Wang B., Song S., Fan Y. (2014). Blue Energy: Current Technologies for Sustainable Power Generation from Water Salinity Gradient. Renew. Sust. Energy Rev..

[10] Logan B.E., Elimelech M. (2012). Membrane-Based Processes for Sustainable Power Generation Using Water. Nature.

[11] Xin W., Zhang Z., Huang X., Hu Y., Zhou T., Zhu C., Kong X.-Y., Jiang L., Wen L. (2019). High-Performance Silk-Based Hybrid Membranes Employed for Osmotic Energy Conversion. Nat. Commun..

[12] Ramon G.Z., Feinberg B.J., Hoek E.M.V. (2011). Membrane-Based Production of Salinity-Gradient Power. Energy Environ. Sci..

[13] Rica R., Ziano R., Salerno D., Mantegazza F., van Roij R., Brogioli D. (2013). Capacitive Mixing for Harvesting the Free Energy of Solutions at Different Concentrations. Entropy.

[14] Brogioli D., Ziano R., Rica R.A., Salerno D., Kozynchenko O., Hamelers H.V.M., Mantegazza F. (2012). Exploiting the Spontaneous Potential of the Electrodes Used in the Capacitive Mixing Technique for the Extraction of Energy from Salinity Difference. Energy Environ. Sci..

[15] Brogioli D., Ziano R., Rica R.A., Salerno D., Mantegazza F. (2013). Capacitive Mixing for the Extraction of Energy from Salinity Differences: Survey of Experimental Results and Electrochemical Models. J. Colloid Interface Sci..

[16] Bijmans M.F.M., Burheim O.S., Bryjak M., Delgado A., Hack P., Mantegazza F., Tenisson S., Hamelers H.V.M. (2012). CAPMIX-Deploying Capacitors for Salt Gradient Power Extraction. Energy Procedia.

[17] Marino M., Kozynchenko O., Tennison S., Brogioli D. (2016). Capacitive Mixing with Electrodes of the Same Kind for Energy Production from Salinity Differences. J. Condens. Matter Phys..

[18] Brogioli D. (2009). Extracting Renewable Energy from a Salinity Difference Using a Capacitor. Phys. Rev. Lett..

[19] Brogioli D., Zhao R., Biesheuvel P.M. (2011). A Prototype Cell for Extracting Energy from a Water Salinity Difference by Means of Double Layer Expansion in Nanoporous Carbon Electrodes. Energy Environ. Sci..

[20] Sales B.B., Saakes M., Post J.W., Buisman C.J.N., Biesheuvel P.M., Hamelers H.V.M. (2010). Direct Power Production from a Water Salinity Difference in a Membrane-Modified Supercapacitor Flow Cell. Environ. Sci. Technol..

[21] Sales B., Burheim O.S., Liu F., Schaetzle O., Buisman C., Hamelers H.V.M. (2012). Impact of Wire Geometry in Energy Extraction from Salinity Differences Using Capacitive Technology. Environ. Sci. Technol..

[22] Hatzell M.C., Cusick R.D., Logan B.E. (2014). Capacitive Mixing Power Production from Salinity Gradient Energy Enhanced through Exoelectrogen-Generated Ionic Currents. Energy Environ. Sci..

[23] Ahualli S., Jiménez M.L., Fernández M.M., Iglesias G., Brogioli D., Delgado Á.V. (2014). Polyelectrolyte-Coated Carbons Used in the Generation of Blue Energy from Salinity Differences. Phys. Chem. Chem. Phys..

[24] Ahualli S., Iglesias G.R., Fernández M.M., Jiménez M.L., Delgado Á.V. (2017). Use of Soft Electrodes in Capacitive Deionization of Solutions. Environ. Sci. Technol..

[25] Kim T., Logan B.E., Gorski C.A. (2017). High Power Densities Created from Salinity Differences by Combining Electrode and Donnan Potentials in a Concentration Flow Cell. Energy Environ. Sci..

[26] La Mantia F., Pasta M., Deshazer H.D., Logan B.E., Cui Y. (2011). Batteries for Efficient Energy Extraction from a Water Salinity Difference. Nano Lett..

[27] Olsson M., Wick G.L., Isaacs J.D. (1979). Salinity Gradient Power: Utilizing Vapor Pressure Differences. Science.

[28] Liu F., Schaetzle O., Sales B.B., Saakes M., Buisman C.J., Hamelers H.V. (2012). Effect of additional charging and current density on the performance of capacitive energy extraction based on Donnan Potential. Energy Environ. Sci..

[29] Tan G., Xu N., Gao D., Zhu X. (2021). Facile Designed Manganese Oxide/Biochar for Efficient Salinity Gradient Energy Recovery in Concentration Flow Cells and Influences of Mono/Multivalent Ions. ACS Appl. Mater. Interfaces.

[30] Zhu X., Yang W., Hatzell M.C., Logan B.E. (2014). Energy Recovery from Solutions with Different Salinities Based on Swelling and Shrinking of Hydrogels. Environ. Sci. Technol..

[31] Arens L., Weißenfeld F., Klein C.O., Schlag K., Wilhelm M. (2017). Osmotic Engine: Translating Osmotic Pressure into Macroscopic Mechanical Force via Poly(Acrylic Acid) Based Hydrogels. Adv. Sci..

[32] Buchholz F.L., Graham A.T. (1997). Modern Superabsorbent Polymer Technology.

[33] Bui T., Cao V.D., Wang W., Kjøniksen A.-L. (2021). Recovered Energy from Salinity Gradients Utilizing Various Poly(Acrylic Acid)-Based Hydrogels. Polymers.

[34] Bui T.Q., Magnussen O.-P., Cao V.D., Wang W., Kjøniksen A.-L., Aaker O. (2021). Osmotic Engine Converting Energy from Salinity Difference to a Hydraulic Accumulator by Utilizing Polyelectrolyte Hydrogels. Energy.

[35] Zavahir S., Krupa I., AlMaadeed S., Tkac J., Kasák P. (2019). Polyzwitterionic Hydrogels in Engines Based on the Antipolyelectrolyte Effect and Driven by the Salinity Gradient. Environ. Sci. Technol..

[36] https://www.chemimpex.com/2-methacryloyloxy-ethyl-dimethyl-3-sulfopropyl-ammonium-hydroxide.

[37] https://www.chemicalbook.com/ChemicalProductProperty_EN_CB7315600.htm.

[38] Khoo S.C., Phang X.Y., Ng C.M., Lim K.L., Lam S.S., Ma N.L. (2019). Recent Technologies for Treatment and Recycling of Used Disposable Baby Diapers. Process Saf. Environ. Prot..

[39] Zhao C., Zhao J., Li X., Wu J., Chen S., Chen Q., Wang Q., Gong X., Li L., Zheng J. (2013). Probing Structure-Antifouling Activity Relationships of Polyacrylamides and Polyacrylates. Biomaterials.

[40] Mary P., Bendejacq D.D., Labeau M.-P., Dupuis P. (2007). Reconciling Low- and High-Salt Solution Behavior of Sulfobetaine Polyzwitterions. J. Phys. Chem. B.

[41] Kasák P., Kroneková Z., Krupa I., Lacík I. (2011). Zwitterionic Hydrogels Crosslinked with Novel Zwitterionic Crosslinkers: Synthesis and Characterization. Polymer.

[42] Ahmad M.B., Huglin M.B. (1994). DSC Studies on States of Water in Crosslinked Poly(Methyl Methacrylate-Co-n-Vinyl-2-Pyrrolidone) Hydrogels. Polym. Int..

[43] Morisaku T., Watanabe J., Konno T., Takai M., Ishihara K. (2008). Hydration of Phosphorylcholine Groups Attached to Highly Swollen Polymer Hydrogels Studied by Thermal Analysis. Polymer.

[44] Bui T.Q., Cao V.D., Wang W., Nguyen T.H., Kjøniksen A.-L. (2021). Energy Lost in a Hydrogel Osmotic Engine Due to a Pressure Drop. Ind. Eng. Chem. Res..

[45] Lin W., Zhang J., Wang Z., Chen S. (2011). Development of Robust Biocompatible Silicone with High Resistance to Protein Adsorption and Bacterial Adhesion. Acta Biomater..

[46] Mohammad S.A., Dolui S., Kumar D., Mane S.R., Banerjee S. (2021). L-Histidine-Derived Smart Antifouling Biohybrid with Multistimuli Responsivity. Biomacromolecules.

[47] Kuang J., Messersmith P.B. (2012). Universal Surface-Initiated Polymerization of Antifouling Zwitterionic Brushes Using a Mussel-Mimetic Peptide Initiator. Langmuir.

[48] Chiao Y.-H., Chen S.-T., Ang M.B.M., Patra T., Castilla-Casadiego D., Fan R., Almodovar J., Hung W.-S., Wickramasinghe R. (2020). High-Performance Polyacrylic Acid-Grafted PVDF Nanofiltration Membrane with Good Antifouling Property for the Textile Industry. Polymers.

[49] Xu J., Lee H. (2020). Anti-Biofouling Strategies for Long-Term Continuous Use of Implantable Biosensors. Chemosensors.

[50] Shao Q., Jiang S. (2015). Molecular Understanding and Design of Zwitterionic Materials. Adv. Mater..

[51] Racovita S., Trofin M.-A., Loghin D.F., Zaharia M.-M., Bucatariu F., Mihai M., Vasiliu S. (2021). Polybetaines in Biomedical Applications. Int. J. Mol. Sci..

[52] Mosnacek J., Osička J., Popelka A., Zavahir S., Ben-Hamadou R., Kasák P. (2018). Photochemical Grafting of Polysulfobetaine onto Polyethylene and Polystyrene Surfaces and Investigation of Long-Term Stability of the Polysulfobetaine Layer in Seawater. Polym. Adv. Technol..

